# Progress in Medicine

**Published:** 1897-01-02

**Authors:** 


### Progress in Medicine.

### DISEASES OF THE NERVOUS SYSTEM.

*(Continued from page 216.)*

**Decussation of the Optic Nerves.**—An investigation which has an interest beyond the limits of the question it was undertaken to settle, viz., the correctness or otherwise of Kolliker's contention that in man there is a complete crossing of the optic nerves at the chiasma, has been reported by L. Jacobsohn,[7] of Berlin. Using Marchi's method of staining, Jacobsohn's method was to extirpate one eye and kill the animal a few weeks later, when degeneration could be easily traced. Rabbits, guinea-pigs, cats, and monkeys were used for experiment. In the rabbits and guinea-pigs no degenerated fibres could be traced through the chiasma to the tract of the same side. All apparently crossed to the tract of the opposite side. But in cats and monkeys, on the other hand, there was a considerable number of fibres to be traced into the optic tract on the same side as the extirpated eye, clearly showing that in these animals the crossing at the chiasma was only an imperfect one. Firstly, there is a strong presumption that partial decussation of the chiasma is the arrangement in man, and Jacobsohn refers to the case shown by Schmidt-Rimpler at the Ophthalmological Congress at Heidelberg, in which such an arrangement was demonstrable. Secondly, it is important to note that we cannot place reliance on the arrangement of nerve distribution in the lower mammals as conclusive evidence of a similar arrangement holding good for human beings, and Jacobsohn's observations show how inconclusive is the evidence of Exner's researches on the laryngeal nerve distribution as being an indication of the conditions normal in mankind.

**Acute Delirium.**—Acute delirium, according to our present conception, is a peri-encephalitis which has a varied etiology, and Warren Babcock[8] finds no less than forty-two alleged predisposing and exciting causes given in text-books as responsible for this disease. Babcock alludes to the recent contribution from H. C. Wood, who makes the following division of cases: (1) Acute peri-encephalitis; (2) an acute affection, primarily centred in the ganglionic cells, but without lesions, that can be demonstrated by our present process. Wood also states that if the observations of Rasori be correct, there must be a third peri-encephalitis, due to the presence of a special organism. Babcock has endeavoured to determine the alleged bacterial nature of the disease. He reports a case of peri-encephalitis in which, on the twenty-ninth day, lumbar puncture was performed for the relief of intracranial pressure, and the fluid was found to contain the micrococcus pneumoniæ crouposæ and streptococcus pyogenes. The patient had had *la grippe* in March, and was admitted to the hospital in May, 1896, incoherent and rambling. He improved after the lumbar puncture on the twenty-ninth day, but subsequently died on the forty-sixth day. A very interesting report of the post-mortem examination, with the chemical and bacteriological examination, and results of inoculation experiments, follows.

**Epilepsy.**—A most important subject is dealt with in a very practical article by Peterson[9] on colonies for epileptics. The author very correctly states at the outset that the moral treatment of epilepsy has scarcely as yet found its way into the text books. Possibly 10 per cent. of all cases of epilepsy become insane, so that they require the custody of an asylum. The remaining 90 per cent. are the cases under consideration in Peterson's article. It is nearly fifty years ago that John Bost began near Bordeaux the system of caring for a variety of chronic cases, inclusive of epileptics, in cottages, grouping them in families, feeling that the true home for such dependents is in the country where they may occupy themselves in the gardens and fields. The little families grew into a happy community—a colony. A colony on somewhat similar lines established at Bielefield, in Westphalia, was visited by Peterson, who was greatly impressed with the happy result. France, Holland, Belgium, and other continental countries have also turned their attention to this class of patients, and, Peterson continues, are following the good example of Germany, which has now a number of colonies for epileptics. In America, the author states that special institutions for epileptics are either established or are actively preparing establishments in Ohio, New York, Pennsylvania, Maryland, Massachusetts, Michigan, Wisconsin, Iowa, Illinois, Virginia, New Jersey, California, Minnesota, and Texas. The tendency of most of them is to follow, as far as possible, some more or less efficient scheme of colonisation. Peterson states that in New York State the number of epileptics immediately available for the purposes of colonisation amount to 1,021, though this total falls short of the actual number who will ultimately become residents of the colony at Craig Colony, and who are truly deserving of being beneficiaries of the State, which he puts at 2,000. At Craig Colony it is the aim of the management to provide out-of-door employment as far as possible for both men and women, feeling that great benefit in the treatment of the disease will be derived from work in the sunshine and open air. Therefore, agriculture, horticulture, floriculture, and market gardening will form a large proportion of the labour of the inhabitants for at least six months of the year. The women will gain great good from employment in raising fruits, flowers, and vegetables.

But in addition to these features which characterise it to a great degree as an agricultural and stock-raising settlement, numerous other trades and callings will need to be summoned into being with the gradual evolution of a self-supporting and independent colony. There must of necessity be masons, painters, carpenters, cabinetmakers, printers, bookbinders, smiths, tailors, shoemakers, and the like; and there will be plenty of indoor work for women in the way of sewing, tailoring, knitting, fancy work, illumination of mottoes, bookbinding, the preserving of fruits, vegetables, seeds, &c. Indeed, the aim is to diversify occupation in every possible way, to consult the patient's own wishes as to his or her special proclivities and abilities, and to make the labour not only of value from the economical standpoint, but also from the standpoint of therapeutics; for it is felt that the exercise, the life out of doors, the manual and industrial training, and the mental occupation will best bring about the bodily and psychic conditions which conduce to improvement and recovery.

It has been found by actual experience in other colonies, and this is already borne out by observations at Craig Colony, that the number of attacks in most patients diminishes after entering upon such colony life; that the patients do not affect each other detrimentally, but that on the contrary each feels that he is on an equality with his associates and no longer isolated, for he is bound together with them by the ties of a common affliction and a common purpose. Out of the negligence, monotony, hopelessness, and often squalor of an almshouse or a wretched home, he comes into the brightness of this new existence. He gains fresh interests, and new hopes and ambitions rouse him from his long apathy. He is made to feel that he may follow the bent of his nature as regards education and occupation, and no longer be debarred from the opportunities for progress in mental development, for recreations and enjoyment, and for social intercourse, so abundantly offered his more fortunate brethren of the outer world.

In addition to the moral therapeutics thus outlined, it is the object of Craig Colony to make every effort to treat each case of epilepsy entrusted to its charge in the best manner possible, in accordance with the latest researches of science in this field, and to carry on original investigations, clinical, chemical, pathological, and therapeutical, with the object constantly in view of discovering the causes of, and best remedies, for the malady. For this purpose chemico-physiological and pathological laboratories are in course of construction.

The colony is designed essentially for State patients, that is, patients upon public charge, but as soon as these have been provided for, private patients will be received whenever there is accommodation for them. There is no restriction as regards admission, except the single one of insanity. Insane epileptics are excluded.

In closing his excellent article, Peterson outlines briefly the main points which need to be considered in planning and organising a colony of this kind.

The value of amyl nitrite in the status epilepticus is well illustrated by a case reported by Robinson.[10] About nine a.m. one day he was called to see a female patient, who had had a succession of fits since half-past eleven p.m. the previous night. She had been an epileptic since childhood, and when Robinson saw her on this occasion he found her lying in a comatose condition, only interrupted about every twenty minutes by an epileptic seizure. The temperature was 103 deg. F., and the pulse rate 130. No appreciable change resulted after ice had been applied to the head and upper part of the spine and a rectal injection of bromide of potassium and chloral hydrate, though this was repeated in two hours' time. Then inhalation of amyl nitrite rendered the fits less severe and less frequent, while the temperature gradually fell. The patient made a rapid recovery. The author states that the effect of the inhalation of the amyl nitrite in this case was most marked, and that it undoubtedly saved her life. He further states that he remembers a similar case treated with amyl nitrite, in which it proved equally efficacious.

**General Paralysis.**—Capps,[11] after a lengthy study of the blood in general paralysis, arrived at the following general conclusions: 1. The hæmoglobin and red corpuscles are always diminished. 2. The specific gravity falls slightly below the normal. 3. Most cases show a slight leucocytosis, amounting on an average to about 22 per cent. above the normal. Early cases may have no leucocytosis whatever. 4. In the differential count a decrease is found in the lymphocytes along with a marked increase in the large mononuclear cells. The eosinophiles in a few cases are very numerous.

In the Convulsions and Apoplectiform Attacks: 1. The red corpuscles and hæmoglobin are usually increased at the time of a convulsion. During an apoplectic attack of long duration they are both somewhat diminished. 2. The specific gravity is variable, sometimes increasing, sometimes diminishing, at the time of an attack. 3. There is a leucocytosis after convulsions and apopletic attacks which is as sudden as it is usually pronounced. It certainly does not appear until within a very short time preceding the convulsion, probably not before it actually takes place. 4. The degree of leucocytosis and the period of its continuance, as a rule, vary directly with the length and severity of the attack, 5. In the production of the leucocytosis the large mononuclear cells are increased relatively more than any other variety. 6. The fact that after convulsions and apoplectic attacks in general paralysis there is not only an increase in the number of white cells, but a change in their character, as shown by the differential count, and at times abnormal cells appear, is an argument against the theory that leucocytosis is merely a change in the distribution of the white corpuscles.

**Disseminated Sclerosis.**—A case, recently reported by Henschen,[12] possesses great interest, inasmuch as the patient contracted diphtheria, followed by indications of widespread involvement of the nerve centres. Post-mortem numerous disseminated sclerotic areas were found throughout the length of the spinal cord. These were few and small in the cervical region, where the postero-median columns had undergone degeneration. The areas of sclerosis were greater in number and extent in the dorsal region, in the lower portion of which the postero-median columns were especially affected; but elsewhere the distribution of the sclerosis was general, though smallest in the pyramidal tracts. In places the grey matter of the cord was invaded. In the affected areas the nerve fibres were more or less completely degenerated; the axis cylinders were best preserved. The separation of healthy from diseased structure was rarely defined sharply. The neuroglia of the white matter appeared increased, and contained many nuclei. In the areas in the grey matter the large ganglion cells presented varying degrees of degeneration, and the tissues contained numerous small glia cells, as well as fibrils. Diphtheria bacilli were looked for, but not found. Degenerative changes were also present in the nerve roots. The vessels were surrounded by an abundant round-cell infiltration; the walls of the vessels had, as a rule, undergone but little change. The nerves of the lower extremities were degenerated, and those of the upper also, though in slighter degree. The brain was not examined.

[7] Neurolog. Central., Sept, 1896; Lancet, Oct. 3, 1896. [8] Med. Rec., Aug. 1, 1896. [9] Med. Rec., Sept. 19, 1896. [10] Lanc., Oct. 3, 1896. [11] Amer. Journ. of Med. Sc., June, 1896. [12] Fortsclin. der. Med., cited by Ed. Med. Rec., Aug. 15, 1896.

